# Acute Gastrointestinal Injury in Critically Ill Patients in a South Indian Intensive Care Unit: A Prospective, Observational, Preliminary Study

**DOI:** 10.7759/cureus.60903

**Published:** 2024-05-23

**Authors:** Dipali A Taggarsi, Sriram Sampath

**Affiliations:** 1 Critical Care Medicine, St. John's Medical College Hospital, Bengaluru, IND

**Keywords:** intra-abdominal pressure, agi grade, feeding intolerance, intensive care unit, critically ill patients, acute gastrointestinal injury

## Abstract

Introduction

The acute gastrointestinal injury (AGI) score was proposed by the Working Group on Abdominal Problems of the European Society of Intensive Care Medicine (ESICM) as a tool to define and grade gut dysfunction. There have not been any studies in India to validate this tool. The objective of this preliminary study was primarily to study the frequency of AGI in the first week of ICU stay in critically ill patients in our intensive care unit (ICU). We also sought to determine the risk factors predisposing to the development of AGI and to determine the prognostic implication of gastrointestinal (GI) injury in critically ill patients.

Materials and methods

A prospective, observational, preliminary, single-center study was conducted on critically ill patients (APACHE II > 8) who were on enteral tube feeds and admitted to a mixed ICU of a tertiary care hospital. Anthropometric data, admission diagnosis, APACHE II score, and comorbidities were recorded. Data of daily heart rate, mean arterial pressure, dose of vasopressors, intra-abdominal pressure, fluid balance, feeding intolerance, mechanical ventilation, and laboratory tests were noted for the first seven days of ICU stay or till ICU discharge, whichever was earlier. The occurrence of AGI score (1-4) during the first seven days of critical illness was the primary outcome of interest. Patient outcome at 28 days was recorded and the impact of the occurrence of AGI on patient outcome was analyzed using the Chi-square test. The patient characteristics associated with AGI were characterized as risk factors and analyzed using a multivariate model.

Results

Data were collected from 33 patients over 201 patient days. The frequency of acute GI dysfunction in the first seven days of ICU stay in our group of patients was 45.45% (15/33). APACHE II, fluid balance, creatinine, and lactate were identified as possible predictors of GI injury based on existing literature. These four variables were entered into an ordinal logistic regression model to assess their ability to predict the occurrence of GI Injury. When fitted into a predictive model, only fluid balance and creatinine were predictive of the final model (p-value < 0.05). A greater fluid balance was predictive in the final model of the development of GI injury; however, it showed negligible clinical significance (OR: 1.00033, 95% CI: 1.000051-1.00061). Lower creatinine levels were predictive in the final model of the development of AGI Injury, as demonstrated by the negative coefficient. Creatinine also had a greater clinical significance (OR: 0.63, 95% CI: 0.44-0.90) in the development of AGI. The impact of the AGI scores on mortality was analyzed. The number of patient days with higher AGI scores was significantly associated with increased mortality at 28 days (p-value < 0.001).

Conclusion

The study showed that nearly half of the critically ill patients included in the study developed acute GI dysfunction. We could not identify any predictors of GI injury based on our results. The result suggested an association between the severity of GI dysfunction and mortality at 28 days.

## Introduction

The gastrointestinal (GI) tract has been increasingly implicated in a role in the initiation and perpetuation of multi-organ dysfunction in critically ill patients. It was more than three decades ago that it was called the “motor of Multiorgan Failure.” This was based on the presence of alterations of gut microflora, the presence of bacteremia in the absence of an identifiable source, and increased mucosal permeability that had been observed in the critically ill [1]. With more and more evidence building up towards the role the gut plays in initiating and perpetuating the disease state, there is a need to clinically assess and define the gut dysfunction that is seen in critically ill patients. There are no readily available markers available to measure the function of the gut, such as creatinine for renal function or a range of biomarkers to assess hepatic function. It has been demonstrated that the presence of GI symptoms such as the absence or abnormality of bowel sounds, the occurrence of vomiting, bowel dilatation, and GI bleeding, especially in the early phase of illness, are associated with poor outcomes in intensive care patients [2,3]. The measurement of intra-abdominal pressure (IAP) is essential to objectively assess the presence of intra-abdominal hypertension and subsequent abdominal compartment syndrome. It has been observed that rising pressures can affect glomerular filtration, cardiac output, and even respiratory movements [4].

Presently, using a combination of clinical signs and IAP, at least 14 scoring systems have been proposed as tools to define and grade gut dysfunction. However, none of these tools have been validated by high-quality studies [5]. One such tool is the acute GI injury (AGI) score which was proposed by the Working Group on Abdominal Problems of the European Society of Intensive Care Medicine (ESICM) [6]. There have been studies that have used the AGI score independently as well as in combination with the SOFA score to assess its influence on outcomes in critically ill patients [7,8]. Despite increasing evidence of its importance, surveys have shown that the monitoring of the GI system and measurement of IAP has been woefully inadequate across intensive care units (ICUs) in the West [9,10]. There is no literature from India investigating the use of the score and its implications in critically ill patients. This was a preliminary study aimed at using the AGI score to assess and grade GI dysfunction in a group of critically ill patients.

## Materials and methods

This was a prospective, observational, preliminary study undertaken in St John’s Medical College Hospital after getting approval from the Institutional Ethics Committee (IEC Study Ref No. 113/2018). Patients admitted to St John’s Medical College Hospital ICU between June 2019 and November 2019 were screened, and those fulfilling the eligibility criteria during the study period were included after obtaining written informed consent from the patient/patient representative. A convenient sampling method was used. As a preliminary study, 33 patients were enrolled in the study. Inclusion criteria included patients above 18 years of age, those expected to stay for at least 48 hours in the ICU, APACHE II > 8, and patients on enteral nutrition (EN) via nasogastric/orogastric tubes. Patients with advanced disease and pregnant patients were excluded from the study. Each patient was screened within 24 hours of admission. Anthropometric data, admission diagnosis, and APACHE II score on the day of admission were recorded. Each patient received standard EN as prescribed by the treating consultant and nutritionist. The gastric aspirate was monitored before each feed. Motility drugs were used at the discretion of the treating consultant. Feeding intolerance (FI) was defined as an inability to achieve a target of 20 kcal/kg body weight/day via the enteral route within 72 h of a feeding attempt or if EN had to be stopped for any clinical reason (vomiting, high GRV, diarrhea, GI bleeding, or presence of entero-cutaneous fistulas). Patient and ventilator data was recorded from the charts. Data of daily heart rate, mean arterial pressure, dose of vasopressors, IAP, fluid balance, FI, mechanical ventilation, and laboratory tests were noted for the first seven days of ICU stay or till ICU discharge, whichever was earlier. The occurrence of AGI score (1-4) during the first seven days of critical illness was the primary outcome of interest. Patient outcome at 28 days was recorded.

Determining the AGI score

The calculation of the AGI score requires information regarding symptoms of GI dysfunction, FI, and IAP values. The presence of any GI symptoms as defined by the ESICM Working Group on Abdominal Problems [6], was also recorded daily. The definitions of GI symptoms are listed in Table 1.

**Table 1:** GI symptoms as defined by the ESICM Working Group on Abdominal Problems GI, Gastrointestinal; cm, centimeter; CT, Computed Tomography; ESICM, European Society of Intensive Care Medicine

GI symptom	Definition
Vomiting (emesis)	Any visible regurgitation of gastric content irrespective of the amount.
Gastric residual volume	Single volume exceeds 200 ml.
Diarrhea	Three or more loose or liquid stools per day with a stool weight greater than 200–250 g/day.
GI bleeding	Any bleeding into the GI tract lumen, confirmed by presence of visible blood in vomited fluids, gastric aspirate or stool.
Paralysis of lower GI tract (paralytic ileus)	Inability of the bowel to pass stool due to impaired peristalsis. The absence of stool for three or more consecutive days without mechanical obstruction is a clinical sign.
Bowel dilatation	If imaging is obtained as determined by the treating consultant, the bowel will be evaluated for dilatation. If colonic diameter exceeds 6 cm (greater than 9 cm for caecum) or small bowel diameter exceeds 3 cm, diagnosed either on plain abdominal X-ray or CT scan, a diagnosis of bowel dilatation was made.
Abnormal bowel sounds	Auscultation for at least 1 minute in two quadrants, done successively (Normal is 5-35/minute).

The initiation of IAP monitoring was based on the discretion of the treating physician. A combination of symptoms of GI dysfunction, FI, and IAP were used to calculate the AGI score daily for every patient during the first seven days of ICU stay. Data was gathered during the first seven days of their ICU stay. The outcome at 28 days was obtained from patient records and/or by telephonic calls.

## Results

Thirty-three patients were recruited, and data was collected over 201 patient days. The mortality at 28 days from ICU admission was used to classify them as survivors and non-survivors (Table 2). There was no loss to follow up.

**Table 2:** Baseline characteristics of survivors and non-survivors among patients included in the study Abbreviations: n (%), number of patients (percentage of patients); SD, Standard Deviation; APACHE, Acute Physiology And Chronic Health Evaluation p-value < 0.05 is statistically significant

Parameter	Outcome at 28 days		P-value
	Survivors	Non-survivors	
Number of patients, n (% of total)	22 (66.67%)	11 (33.33%)	
Age in years, Mean+SD	52.45 + 6.94	42.63 + 11.39	p = 0.11
Sex			p = 0.33
Male, n (%)	19 (57.58%)	8 (24.24%)	
Female, n (%)	3 (9.09%)	3 (9.09%)	
APACHE II, Mean + SD	17.54 + 2.58	18.36 + 5.13	p = 0.73
Mechanically ventilated, n (% of survivors / non-survivors)	16 (72.7%)	10 (90.9%)	p = 0.23
Vasopressor use, n (% of survivors / non-survivors)	8 (36.4%)	11 (100%)	p = 0.0004

Of the 33 patients, 27 (81.82%) were male and six (18.18%) were female. The mean age among survivors and non-survivors was 52.45 + 6.94 years versus 42.63 + 11.39 years (p > 0.05). The mean APACHE II scores of the survivors and non-survivors were 17.54 ± 2.58 (mean ± SD) and 18.36 ± 5.13 (mean ± SD) respectively. There was no significant difference in the APACHE II score between survivors and non-survivors (p = 0.73). Out of the 22 survivors, 16 patients (72.7%) were ventilated and out of 11 non-survivors, 10 patients (90.9%) were ventilated. There was no significant difference in mechanical ventilation between the two groups (p > 0.05). There was a significant difference in the frequency of vasopressor use between survivors and non-survivors (p < 0.05), with increased vasopressor use among non-survivors.

Of the data collected, 15 (45.45%) patients showed evidence of gut dysfunction during their ICU stay. The number of patients with AGI scores of 1, 2, and 3 were two (6.06%), 11 (33.33%) and two (6.06%), respectively. There were no patients with AGI grade 4. The primary outcome was the occurrence and severity of AGI in the first week of ICU stay. The maximum AGI score during the week was noted for each patient. Of a total number of 201 documented patient days, the Chi-square test was used to assess the severity of AGI between survivors and non-survivors at 28 days following ICU admission. Higher AGI score days individually were significantly associated with increased mortality at 28 days (p < 0.0009) (Table 3).

**Table 3:** Primary outcome of patients with their daily AGI scores Abbreviations: AGI, Acute Gastrointestinal Injury; N (%), Number of patient days (Percentage of total patient days) p-value < 0.05 is statistically significant

Daily AGI score	Survivors, N (%)	Non-survivors, N	p-value
0	122 (60.70%)	38 (18.91%)	<0.0009
1	2 (1%)	0	
2	16 (7.96%)	13 (6.46%)	
3	0	10 (4.97%)	
4	0	0	

The maximum AGI score in the first week of illness was also compared between the two groups, but it showed no significant correlation to mortality at 28 days (Table 4).

**Table 4:** Differences in maximum AGI score between survivors and non-survivors Abbreviations: AGI, Acute Gastrointestinal Injury; n (%), Number of patients (percentage of total patients) p-value < 0.05 is statistically significant

Maximum AGI score	Survivors, n (%)	Non-survivors, n (%)	p - value
0	14 (42.42%)	4 (12.12%)	p = 0.081
1	2 (6.06%)	0	
2	6 (18.18%)	5 (15.15%)	
3	0	2 (6.06%)	

We also sought to look for associations between AGI and certain risk factors for the same. Of the parameters collected, APACHE II [11-13], fluid balance [13-15], creatinine [13,16], and lactate [13,14] were chosen based on existing meta-analysis, clinical guidelines, and clinical judgment. They were entered into an ordinal logistic regression model to assess their ability to predict the occurrence of AGI. In the proportional odds model, only fluid balance and creatinine were significant predictors of success (p < 0.05). The model showed that increasing fluid balance is associated with an increased chance of developing AGI (Table 5). However, the odds of this were extremely small and clinically insignificant (OR: 1.00033, 95% CI: 1.00005-1.00061). Creatinine was a more clinically significant co-variate in the predictive model, and it demonstrated an inverse relationship with the development of AGI (regression co-efficient: -0.46, 95% CI: -0.78 to -0.02) with greater odds (OR: 0.63, 95% CI: 0.44-0.90) (Table 5).

**Table 5:** Application of ordinal logistic regression analysis in determining patient characteristics associated with development of acute GI Injury Abbreviations: CI, Confidence Interval Lactate and APACHE II did not have any predictive value. p-value < 0.05 is statistically significant

Co-variable	Regression coefficient (CI)	Standard error	P-value	Odds ratio (CI)
Fluid Balance	0.00033 (0.00005 - 0.00060)	0.00014	0.02	1.00033 (1.00005 - 1.00061)
Creatinine	-0.46 (-0.78 - -0.02)	0.18	0.01	0.63 (0.44 - 0.90)

## Discussion

This was a single-center, observational, descriptive, preliminary study done to assess acute GI dysfunction among patients admitted to a mixed ICU. The patient cohort was predominantly medical, with only two of the patients in the study admitted for surgical causes. Of the total number of patients recruited, 45.45% of the patients developed AGI of grades ranging from 1 to 3. Previous studies have noted incidence rates ranging from 46% to 100% [7,12,17].

There was no significant difference between survivors and non-survivors with respect to age, sex, or APACHE II scores. While discussing GI dysfunction, the focus thus far has been predominantly on intra-abdominal hypertension which is one of the objectively measurable parameters to diagnose and determine the severity of AGI [10,14,15,18]. Experimental studies have demonstrated that intra-abdominal hypertension can significantly reduce mesenteric blood flow in the intestinal mucosa, increase intestinal permeability, result in endotoxemia, and lead to irreversible damage to the mitochondria and necrosis of the gut mucosa [19]. Whereas some investigators have strongly advocated the need for early measurement and targeted therapy [10], some have questioned the prudence of measuring it in every patient [14]. A study by Cheatham et al. showed that protocolized ICU management which included an open abdomen significantly improved survival as opposed to patients without an open abdomen. However, this was an observational study and there was no randomization. Thus, not much could be said conclusively about its impact on mortality [20]. There exist patient groups that benefit from IAP monitoring such as those with acute pancreatitis, abdominal surgeries, and burns requiring high-volume resuscitation [18,21,22]. There are still grey areas regarding determining an appropriate group of patients who would benefit from regular measurement of IAP. In our study, only five patients underwent IAP measurement. This may have compromised the interpretation, as the definition of AGI relied heavily on clinical symptomatology, FI, and radiological findings. It is likely that several patients without overt signs of intra-abdominal hypertension may have been missed altogether.

In our group of patients, APACHE II could not predict the occurrence of AGI. This differs from the findings of Li et al. [11] and Zhang et al. [12], where a higher APACHE II score correlated with a higher AGI score.

The impact of AGI on 28-day mortality was assessed in this cohort. Studies have shown that a higher AGI score is associated with greater 28-day mortality [7,11,12,23]. In our study, the maximum AGI score recorded in the first week was not associated with an increased 28-day mortality. However, higher daily AGI scores on a greater number of days were associated with higher mortality.

We also studied the factors associated with the occurrence of AGI. A number of studies have attempted to identify the characteristics predisposing to elevated IAP [15,24]. A meta-analysis in 2015 revealed that across the general patient population, large-volume resuscitation, markers of shock and hypotension, and respiratory status were common risk factors for IAH and ACS. Elevated creatinine and increased disease severity scores were found to have an association with IAH and ACS in patients with acute pancreatitis [13]. 

The results suggested that increased fluid balance may result in AGI. This is in concordance with the findings of other studies [11,24]. However, we found that this association was extremely weak. (OR: 1.00033, 95% CI: 1.00005 - 1.00061). Our study did not demonstrate a clinically significant relationship between fluid balance and the development of AGI, essentially because it was not powered to determine an association.

There was paradoxically a negative relationship between AGI scores and serum creatinine levels (OR 0.67 95%CL: 0.46-0.97) which was contradictory to existing evidence regarding the association of higher IAP levels and onset of renal dysfunction characterized by elevated serum creatinine [13]. This could likely be explained by the low percentage of patients who underwent IAP monitoring, thus, resulting in an underestimation of AGI in these patients. We also hypothesize that patients admitted with higher serum creatinine levels were likely to receive fluids more cautiously and hence were less likely to develop Intra-abdominal hypertension and subsequent AGI. The study was not powered to establish an association between serum creatinine and AGI.

Limitations

The percentage of patients developing AG in the data set, ranging from grades I-III, was 45.45%. This does not represent the actual incidence in our ICU population. An estimation of the incidence required consecutive sampling of patient data. However, logistical issues resulted in an inability to conduct consecutive sampling. Hence, the data did not allow for the incidence of AGI to be calculated among our cohort.

Exclusion of patients who did not stay > 48 hours means there was a pre-selection bias. It is possible that a combination of the least and the most critical patients were excluded from the study. This could also explain why APACHE II had no association with mortality. Reintam et al. reported a similar outcome [25].

Only a small fraction of the patients underwent IAP monitoring. The risk factors for elevated IAP are exhaustive, however, poorly substantiated. This, however, compromises the definition of AGI that was used, as IAP is a part of it. Several patients with occult elevation in IAP may have been missed from the study, thus, missing a lot of valuable information. An additional confounder could be that patients with obvious abdominal pathologies or distension were more likely to undergo IAP monitoring. Thus, it is possible that this contributed to higher AGI grade days amongst non-survivors. We found the inconsistencies in IAP measurement to be one of the biggest deterrents against conducting observational studies using the WSACS definition of AGI.

This is only a single-center preliminary study. The study was not powered to identify the risk factors of AGI or its association with 28-day mortality.

Future directions

The present observational study design used in our study cannot be used in our set-up to evaluate AGI as only a small number of our patients undergo IAP monitoring. A larger study outlining clear indications for such monitoring is needed to determine the true prevalence of AGI and its impact on patient outcomes. Moreover, we need to evaluate whether determining this score has any impact on patient management and whether there is a change in course of treatment leading to better patient outcomes.

## Conclusions

The study showed that nearly half of the critically ill patients who were included in the study developed AGI. The result suggested an association between the severity of GI dysfunction and mortality at 28 days. Patients with a positive fluid balance were found to be more likely to develop AGI. The strength of this relationship was weak and not clinically significant. Larger studies with clearly defined indications for IAP monitoring are needed to identify the risk factors of AGI as well as its association with patient outcomes.
